# Multiple-Tissue and Multilevel Analysis on Differentially Expressed Genes and Differentially Correlated Gene Pairs for HFpEF

**DOI:** 10.3389/fgene.2021.668702

**Published:** 2021-07-08

**Authors:** Guofeng Zhou, Shaoyan Sun, Qiuyue Yuan, Run Zhang, Ping Jiang, Guangyu Li, Yong Wang, Xiao Li

**Affiliations:** ^1^First Clinical Medical College, Shandong University of Traditional Chinese Medicine, Jinan, China; ^2^School of Mathematics and Statistics, Ludong University, Yantai, China; ^3^CEMS, NCMIS, MDIS, Academy of Mathematics and Systems Science, Chinese Academy of Sciences, Beijing, China; ^4^School of Mathematical Sciences, University of Chinese Academy of Sciences, Beijing, China; ^5^Department of Cardiovascular, Affiliated Hospital of Shandong University of Traditional Chinese Medicine, Jinan, China

**Keywords:** heart failure with preserved ejection fraction, differentially expressed genes, differentially correlated gene pairs, differential network, molecular docking

## Abstract

Heart failure with preserved ejection fraction (HFpEF) is a complex disease characterized by dysfunctions in the heart, adipose tissue, and cerebral arteries. The elucidation of the interactions between these three tissues in HFpEF will improve our understanding of the mechanism of HFpEF. In this study, we propose a multilevel comparative framework based on differentially expressed genes (DEGs) and differentially correlated gene pairs (DCGs) to investigate the shared and unique pathological features among the three tissues in HFpEF. At the network level, functional enrichment analysis revealed that the networks of the heart, adipose tissue, and cerebral arteries were enriched in the cell cycle and immune response. The networks of the heart and adipose tissues were enriched in hemostasis, G-protein coupled receptor (GPCR) ligand, and cancer-related pathway. The heart-specific networks were enriched in the inflammatory response and cardiac hypertrophy, while the adipose-tissue-specific networks were enriched in the response to peptides and regulation of cell adhesion. The cerebral-artery-specific networks were enriched in gene expression (transcription). At the module and gene levels, 5 housekeeping DEGs, 2 housekeeping DCGs, 6 modules of merged protein–protein interaction network, 5 tissue-specific hub genes, and 20 shared hub genes were identified through comparative analysis of tissue pairs. Furthermore, the therapeutic drugs for HFpEF-targeting these genes were examined using molecular docking. The combination of multitissue and multilevel comparative frameworks is a potential strategy for the discovery of effective therapy and personalized medicine for HFpEF.

## Introduction

Heart failure with preserved ejection fraction (HFpEF) is a common coronary disease characterized by left ventricular diastolic dysfunction, unimpaired left ventricular ejection fraction, and cardiac remodeling (Cuijpers et al., 2020). The mechanisms underlying HFpEF have not been completely elucidated, which has limited the development of effective drugs for HFpEF (Roh et al., 2017). HFpEF is a complex disease involving multiple genes and interactions between the adipose tissue, heart, and cerebral arteries (Borlaug, 2014; Altara et al., 2017; Cogswell et al., 2017; Kitzman and Nicklas, 2018). The interaction between tissues, which are involved in various biological functions, is involved in the pathogenesis of HFpEF. Thus, analyzing the shared and unique pathological features of these three tissues in HFpEF at the molecular level can aid in elucidating the underlying mechanisms and therapeutic targets.

The methods used in systems biology and high-throughput techniques have successfully reconstructed various disease-related networks for pathological conditions, such as cancer, type 2 diabetes mellitus (T2DM), and influenza. These methods enable the integration and interpretation of functional genomic datasets and the identification of novel biomarkers or modules, which can aid in the elucidation of the molecular mechanisms of diseases (Tang et al., 2018; Sun et al., 2019b; Wu Y. et al., 2020). In particular, multilevel analysis based on tissue-related networks can systemically reveal the pathophysiology of the disease through the integration of several target tissues and the identification of key pathways or biomarkers (Knaack et al., 2014; Sun et al., 2019a). Molecular docking can identify therapeutic drugs based on the results of network analysis (Gao et al., 2020). In particular, molecular docking can potentially identify therapies and enable the development of personalized medicine. Furthermore, molecular docking can aid in the discovery of the binding sites of molecular compounds in key genes and the elucidation of the molecular effects of therapeutic agents on key genes.

**FIGURE 1:**
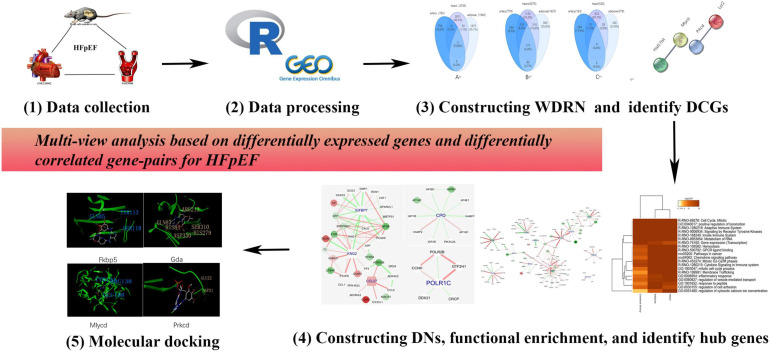
The flowchart of this research. We proposed a multiple-tissue and multilevel comparative framework based on differentially expressed genes and differentially correlated gene pairs for heart failure with preserved ejection fraction (HFpEF).

Here, we propose a multitissue and multilevel comparative framework to identify effective therapies and personalized medicine for HFpEF. A detailed flowchart (drawn using Pathway Builder Toll 2.0) is shown in Figure 1. In addition to the differentially expressed genes (DEGs), we propose differentially correlated gene pairs (DCGs) to obtain multigene information. A wild-type disease-related network (WDRN) was generated by integrating DEGs and protein–protein interaction (PPI) networks. Next, an intensive-type disease-related network was generated based on the identified DCGs. The network structure was explored by extracting densely connected network components using the molecular complex detection (MCODE) algorithm in the merged network of the three tissue-specific disease-related networks. Thus, we performed a comprehensive analysis at the gene, network, and module levels and presented the shared and unique pathophysiological features of the cerebral artery, adipose tissue, and heart in HFpEF. The Comparative Toxicogenomics Database (CTD) was used to verify the results of this study. The hub genes characterized in this study were closely related to HFpEF in humans. This indicated that these genes are effective personalized therapeutic targets that can indicate the efficacy of HFpEF treatment. Finally, molecular docking was performed to identify personalized therapeutic agents for HFpEF.

## Materials and Methods

### Data Collection

Twelve specific pathogen-free male Dahl salt-sensitive rats (aged 6-week-old and weighing 160–180 g) (Certificate No. 2016-0006) were obtained from the Charles River Animal Laboratory (Beijing, China). The rats were housed under the following controlled conditions: circadian conditions, 12-h dark/light cycle; temperature, 20–24°C; relative humidity, 40–60%; noise levels, ≤ 60 dB. The rats were randomly divided into the following two groups (six rats/group): HFpEF group, fed on chow supplemented with 8% NaCl for 11 weeks, and control group, fed on chow supplemented with 0.3% NaCl for 11 weeks. The adipose tissue was collected from the HFpEF and control groups after 11 weeks. All experiments were approved by the Animal Ethics Committee of the Shandong University of Traditional Chinese Medicine (Ethics No. SDUTCM2018071501). The total RNA was extracted from the samples and quantified. Oligo (dT) magnetic beads were used to enrich the messenger RNA (mRNA). The complementary DNA (cDNA) library was quantified using a Qubit Fluorometer, hermo Fisher Scientific, United States. The insert size of the cDNA library was analyzed using an Agilent 2100 bioanalyzer (Agilent Technologies, Sta. Clara, CA, United States). Finally, the cDNA was sequenced using the synthesis method. The gene expression profiles of the heart (accession ID: GSE126062) and cerebral artery (accession ID: GSE5488) were obtained from the Gene Expression Omnibus database^1^. The GSE126062 and GSE5488 datasets contained six samples (three healthy samples and three HFpEF samples).

### mRNA Sequencing

RNA degradation and contamination was monitored on 1% agarose gels. A NanoPhotometer^®^ spectrophotometer (IMPLEN, Westlake Village, CA, United States) was used to check the purity of RNA. RNA integrity was checked by RNA Nano 6000 Assay Kit of the Bioanalyzer 2100 system (Agilent Technologies). A total amount of 1 μg RNA per sample was regarded as RNA sample for mRNA sequencing. Poly-T oligo-attached magnetic beads was used to obtain total mRNA from the RNA sample. M-MuLV Reverse Transcriptase (RNase H-) and random hexamer primer were used to obtain the first-strand cDNA. DNA Polymerase I and RNase H were used for second-strand cDNA synthesis. cDNAs of 250–300 bp were selected, and purified by AMPure XP system (Beckman Coulter, Beverly, MA, United States). USER Enzyme (NEB, Ipswich, MA, United States) was used to the adaptor-ligated cDNA. Adaptor-ligated cDNA, Phusion High-Fidelity DNA polymerase, and Universal PCR primers were used for PCR. In addition, the AMPure XP system, Beckman Coulter, USA was used to purify the PCR products, and library quality was assessed by Agilent Bioanalyzer 2100 system. The clustering of the index-coded samples was performed on a cBot Cluster Generation System using TruSeq PE Cluster Kit v3-cBot-HS (Illumina, San Diego, CA, United States) according to the manufacturer’s instructions. After cluster generation, the library preparations were sequenced on an Illumina Hiseq platform, and 125 bp/150 bp paired-end reads were generated.

### Data Processing

The raw data in the GSE126062 and GSE5488 datasets were subjected to probe summarization and logarithmic transformation. The expression matrix was constructed using the robust multiarray average, and the probe ID was converted to a gene symbol. The DEGs in the heart, adipose tissue, and cerebral arteries were identified using the R package “limma” with the following parameters: *p* < 0.05 and | log_2_ fold change (FC)| > 0.5. The correlation between co-DEPs and cardiovascular diseases was examined using CTD^2^.

### Construction of WDRN

The PPI network was downloaded from STRING (version 11.0^3^). We first downloaded the PPI network scored links between proteins from STRING (version 11.0, see text footnote 3) and reserved the interactions with scores above 900 at a confidence level (Sharma and Colonna, 2021). The edges were removed such that the vertices did not comprise DEGs. The DEGs and associated genes were identified, and a network was constructed with vertices comprising either DEGs or genes that directly interacted with a DEG. This network contained the molecular and functional factors perturbed in the disease condition as a wild-type control and was named WDRN.

### Identification of DCGs

The disease information was extracted from the constructed WDRN based on the disruption of PPI in the disease dataset. The interaction between genes or gene products (such as proteins) can be measured using the correlation coefficient. Thus, the disruption of one interaction in the disease dataset results in a change in its correlated interaction. If two gene expression vectors from a gene pair were highly correlated under a disease condition but were uncorrelated or exhibited low correlation under healthy conditions, they were defined as DCGs. The DCGs were considered disordered interactions caused by the disease.

To effectively measure the correlation of a gene pair in the datasets of three to five samples in each tissue, the correlation coefficient was considered, and the hypothesis was tested. Heterogeneity among expression profiles cannot be ruled out, as they were derived from multiple tissues and different platforms. Spearman’s correlation coefficient was used to measure the strength of the correlation between paired data. For the two random variables *X* = *x*_1_,*x*_2_,…,*x*_*n*_ and *Y* = *y*_1_,*y*_2_,…,*y*_*n*_, Spearman’s correlation calculates Pearson’s correlation based on the ranked values of the data. If *u*_1_,*u*_2_,…,*u*_*n*_ denote the ranks of the *n* observed values of *X* and *v*_1_,*v*_2_,…,*v*_*n*_ denote the ranks of the *n* observed values of *Y*, Spearman’s correlation coefficient is defined by the following equation:

$$r_{s}=\frac{S_{u\,v}}{\sqrt{S_{u}^{2}\,S_{v}^{2}}}$$

where *r*_*s*_ is the Spearman’s correlation coefficient, *S*_*uv*_ is the sample covariance between *u*′s and *v*′s, $S_{u}^{2}$ and $S_{v}^{2}$ are the sample variances of *v’*s and *u’*s, respectively (Looney and Hagan, 2011).

Based on Spearman’s correlation coefficient, a significance test was performed to determine the linear correlation in *X* and *Y*. The correlation of a gene pair was considered significant at *p* < 0.05.

### Molecular Docking

The Drugbank database^4^ was used to identify small-molecule compounds that target the genes observed in the identified MCODE components. The Protein Data Bank^5^ was used to determine the protein structures of these gene products. Water molecules and eutectic ligands were deleted from protein, and side chain fixation, residue repair, and hydrogenation also contribute to protein preparation. Molecular docking was performed in Surflex-Dock module of SYBYL 2.1. Energy optimization was carried out in AMBR7, and automatic mode was used to obtain the active pockets. In addition, the parameters for SYBYL 2.1 were set as default. The scores of molecular docking reflect the strength of the interactions between proteins and molecules.

## Results

### Capturing the Dysfunctional Information

#### Identification of DEGs

**FIGURE 2:**
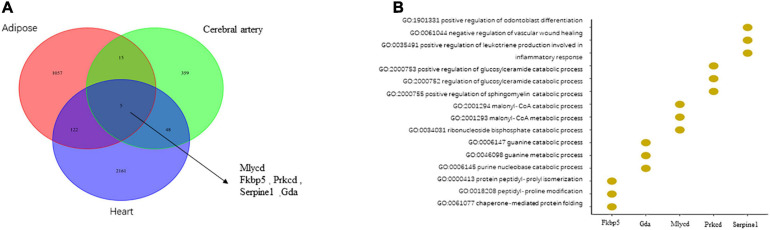
The comparison of differentially expressed genes (DEGs) in the cerebral artery, heart, and adipose and biological processes related to housekeeping genes. **(A)** The comparison of DEGs in the heart, adipose, and cerebral artery. **(B)** The biological processes related to housekeeping genes.

In total, 427, 2,336, and 1,199 DEGs were identified in the cerebral artery, heart, and adipose tissue of the HFpEF group, respectively. Among these three tissues, only the following five housekeeping genes were identified: *MLYCD* (malonyl-CoA decarboxylase), *FKBP5* (FKBP prolyl isomerase 5), *PRKCD* (protein kinase C, delta), *SERPINE1* (serpin family E member 1), and *GDA* (guanine deaminase) (Figure 2A). The Venn diagram of DEGs among the three tissues and the biological process annotation for the five housekeeping genes provided a general and simple illustration of the pathophysiology of HFpEF (Figure 2B). For example, the biological processes of *MLYCD*, *FKBP5*, *PRKCD*, and *GDA* were associated with energy metabolism and immune response, while those of *SERPINE1* were associated with the inflammatory response. This was consistent with the pathological phenotypes, including immune dysregulation, metabolic dysfunction, and activation of chronic inflammation, of various tissues in patients with HFpEF (Hage et al., 2020; Sava et al., 2020; Schiattarella et al., 2021).

#### Identifying DCGs

In addition to individual genes, gene interactions are highly specific to the disease, as the functions of genes and gene products are dependent on biochemical or physical interactions. We assumed that molecular interactions are perturbed by genetic or epigenetic factors that lead to molecular dysfunctions associated with human diseases. To identify these interactions, a WDRN was constructed for each target tissue by integrating the DEGs and PPI network from STRING. For every edge, at least one vertex comprising DEGs between healthy and disease conditions was incorporated in the WDRN.

The tissue-specific WDRN can indicate global changes in the disease. The WDRN of the cerebral artery comprised 14,372 edges and 2,612 nodes encompassing 267 DEGs (10.22%). Meanwhile, the WDRN of the heart comprised 46,972 edges and 5,195 nodes comprising 859 DEGs (16.53%). Furthermore, the WDRN of the adipose tissue comprised 25,030 edges and 4,631 nodes encompassing 560 DEGs (12.09%).

**FIGURE 3:**
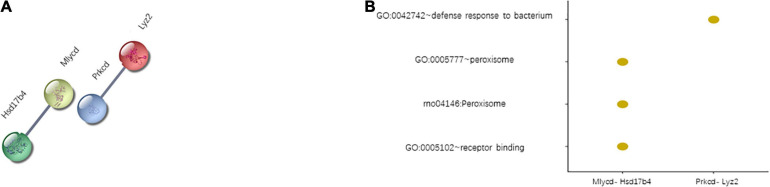
Two housekeeping differentially correlated gene pairs (DCGs). **(A)** List of housekeeping DCGs. **(B)** Function enrichment results of housekeeping DCGs.

For each tissue, the pathological interactions were captured based on the WDRN. Specifically, if two gene expression vectors from a gene pair were highly correlated under one condition (healthy or diseased condition) but uncorrelated or weakly correlated under the other condition (diseased or healthy condition), this gene pair was described as a DCG. The DCG interaction was considered a pathological interaction. In total, 783, 2,706, and 1,945 DCGs were identified for the cerebral artery, heart, and adipose tissue, respectively. Only *PRKCD*-*LYZ2* and *MLYCD*-*HSD17B4* (Figure 3A) were housekeeping DCGs for the three target tissues. The interaction between *MLYCD* and *HSD17B4* influences fatty acid oxidation. Additionally, *MLYCD* and *HSD17B4*, which play an important role in mitochondrial function, lipid metabolism, and endothelial structure, are expressed in the cerebral artery and adipose tissue (Gautier et al., 2015; Chaanine et al., 2019; Jo et al., 2020). The *PRKCD*–*LYZ2* pair is reported to be strongly correlated with the inflammation response (Xu et al., 2014) and may promote the activation of chronic systemic inflammation. These two housekeeping DCGs are closely correlated with HFpEF. However, further studies are needed to elucidate the specific correlation. An elementary functional analysis was performed for these two DCGs; the results are shown in Figure 3B. In *Potential drug candidates by molecular docking for housekeeping DEGs and DCGs*, we will further discuss drug discovery and molecular docking.

#### Constructing Differential Networks

For each tissue, the identified DCGs constitute an intensive-type disease-related network, which was termed differential network (DN). DN can characterize the phenotypic difference between the healthy and disease states at both molecular and interaction levels. In DN, each edge indicates the differential correlation of the molecular interaction between healthy and disease conditions. Each node or its linked node corresponds to the DEG or its product between healthy and diseased conditions. Thus, the DN could provide comprehensive and detailed information on HFpEF.

The cerebral artery DN comprised 783 edges and 779 nodes encompassing 163 DEGs (20.92%). The heart DN comprised 2,706 edges and 2,075 nodes encompassing 520 DEGs (25.06%), while the adipose tissue DN comprised 1,945 edges and 1,627 nodes encompassing 379 DEGs (23.29%). The coverage rate of DEGs in DN was higher than that in WDRNs. These results demonstrate that WDRN is a wild-type disease network, whereas DN is an intensive-type network.

**FIGURE 4:**
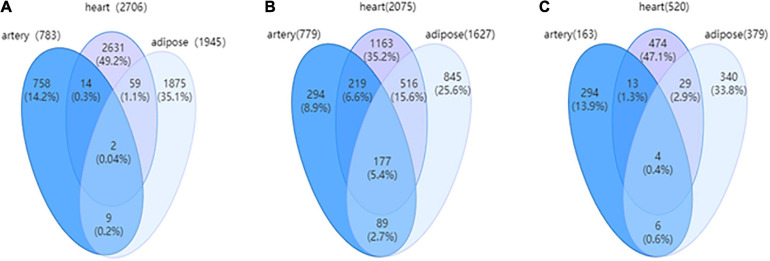
**(A)** The Venn diagram for edges among three tissue-dependent differential networks (DNs). **(B)** The Venn for nodes among three tissue-dependent DNs. **(C)** The Venn for differentially expressed genes (DEGs) covered by three tissue-dependent DNs.

The relevance of the three targeted tissues of HFpEF can be further investigated based on DNs. Figure 4 shows the Venn diagrams of the three tissue-specific DNs with edges, nodes, and covered DEGs. The edges, which indicate the differential interactions, were significantly different between the three tissues. Additionally, the DNs of the heart and adipose tissues exhibited high similarity. In addition, the characteristics of three tissue-specific differential networks are shown in Supplementary Table 1.

### Functional Enrichment Analysis

#### Functional Enrichment Analysis of Nonspecific Genes

**FIGURE 5:**
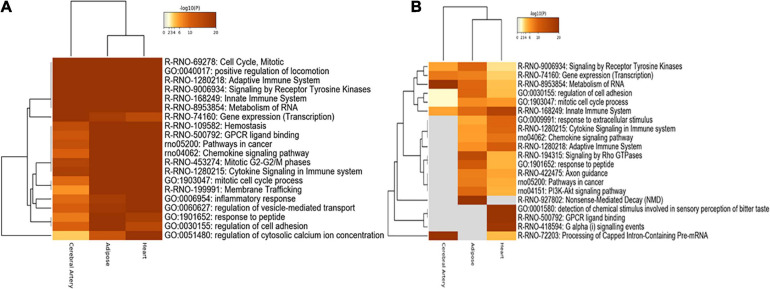
Functional enrichment analysis. **(A)** Functional enrichment analysis of nonspecific genes. **(B)** Functional enrichment analysis of tissue-specific genes.

Functional enrichment analysis of nonspecific genes in DN can indicate their relevance in the tissues. Consistent with the number of overlapping genes and interactions, the aberrant biological processes in the heart were similar to those in the adipose tissue. Shared biological processes among the cerebral artery, heart, and adipose tissue were closely related to cell cycle, mitosis, positive regulation of locomotion, adaptive immune system, signaling receptor tyrosine kinases, innate immune system, metabolism of RNA, and chemokine signaling pathway. The most common biological processes, including hemostasis, G-protein coupled receptor (GPCR) ligand binding, cancer-related pathway, mitotic G2–G2M phases, cytokine signaling in the immune system, and membrane trafficking, between the adipose tissue and heart were considered to be associated with HFpEF. For example, the cancer-related pathways were enriched. Cancer is a common comorbidity of HFpEF. Additionally, the treatment strategies for cancer increase the risk of HFpEF (Lund et al., 2014; Saiki et al., 2017). Gene expression (transcription) process was markedly enriched in the cerebral artery. The inflammatory response, regulation of vesicle-mediated transport, and regulation of cytosolic calcium ion concentration biological processes were specifically enriched in the heart. The response to peptides and regulation of cell adhesion were enriched in the adipose tissue (Figure 5A).

#### Functional Enrichment Analysis of Tissue-Specific Genes

The functional enrichment of tissue-specific genes was explored to identify the biological processes specific to each tissue. Consistent with the functional enrichment analysis results of nonspecific genes, the metabolism of RNA and processing of capped intron-containing pre-mRNA were highly enriched in the cerebral artery. Furthermore, rho GTPase signaling, response to peptides, and nonsense-mediated decay were significantly enriched in the adipose tissue, while other biological processes were similar to those obtained in the nonspecific gene enrichment analysis.

The innate immune system, response to extracellular stimuli, cytokine signaling in the immune system, adaptive immune system, detection of chemical stimuli involved in sensory perception of bitter taste, GPCR ligand binding, and G alpha signaling were highly enriched in the heart (Figure 5B).

### Identifying Hub Genes in the Network

#### Hub Genes of Tissue-Specific Genes

Next, the tissue-specific hub genes were captured in the networks. Tissue-specific MCODE components were first divided based on the tissue-specific edges, and the components score higher than 3 were reserved in our study. MCODE algorithm could detect densely connected regions in large protein–protein interaction networks that may represent molecular complexes; it owns the advantage over other graph clustering methods of having a directed mode that allows fine tuning of clusters of interest without considering the rest of the network and allows examination of cluster interconnectivity, which is relevant for protein networks (Bader and Hogue, 2003). Three MCODE components were obtained for each tissue sample. The specific genes in these nine MCODE components were analyzed, and the genes with a score higher than 10 were selected as tissue-specific hub genes. In the cerebral artery, none of the genes exhibited a score higher than 5. Hence, *POLR1C* with a score of 5 was selected as the artery-specific hub gene. One, one, and three hub genes were identified for the cerebral artery, heart, and adipose tissue, respectively.

**FIGURE 6:**
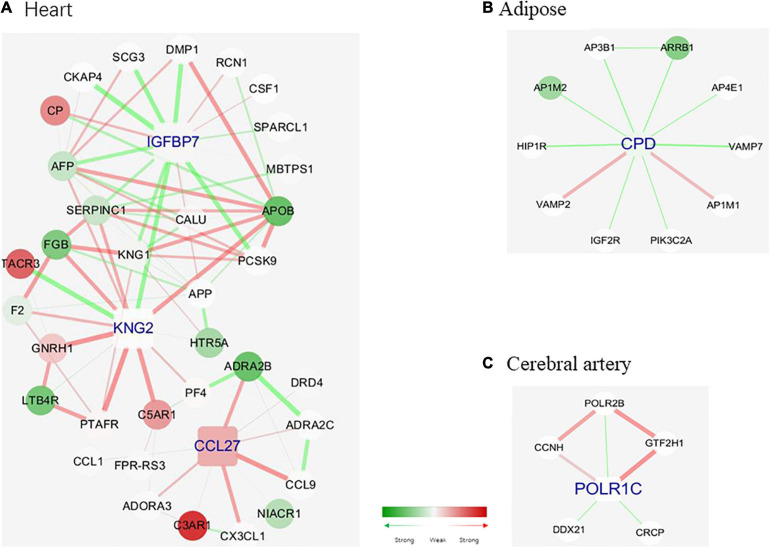
Tissue-specific hub genes and their corresponding modules. **(A)** IGFBP 7, KNG 2, and CCL 27. **(B)** CPD. **(C)** POLR1C. Gene colored in green means differential expression between disease and health conditions and was significantly downregulated under disease, while red means upregulated. One edge colored with green means the interaction between its two linked genes was perturbed and was inactivated under disease condition, while red means activated. The width of edges reflects the magnitude of the perturbation. Tissue-specific hub genes were shaped with round rectangle and labeled with blue.

Figure 6 shows the hub genes and their first neighbors in the network. The hub genes exhibited tissue-specific expression. The tissue-specific hub genes are associated with specific biological functions of each tissue. *IGFBP7* (insulin-like growth factor binding protein 7), *KNG2* (kininogen 2), and *CCL27* (C–C motif chemokine ligand 27) were the heart-specific hub genes. Previous studies have reported that the expression level of *IGFBP7* is associated with diastolic function and exercise capacity in patients with HFpEF (Gandhi et al., 2016). The interaction between *IGFBP7* and *KNG2* was associated with the biological processes of the extracellular space and the severity of myocardial fibrosis in patients with HFpEF (Rommel et al., 2017). Additionally, the enhanced interaction between *KNG2* and *C5AR1* (C5a anaphylatoxin chemotactic receptor 1) promoted the expression of C5AR1 and activated chronic inflammation in the heart. Most edges of the adipose-tissue-specific network were linked to the hub gene *CPD* (carboxypeptidase D), which is involved in the posttranslational processes and influences the activation of peptides (Xin et al., 1997). Changes in the strength of the interactions between *CPD* and *ARRB 1* (*Arrestin Beta 1*) may inhibit the activation of ARRB 1 and consequently affect the posttranslational processes (Xu et al., 2020). *POLR1C* (RNA polymerase I and III subunit C), which was the cerebral artery-specific hub gene, plays an important role in ribosome biogenesis (Noack Watt et al., 2016). Consistent with the biological function of *POLR1C*, genes linked to POLR1C exerted similar effects (Fujii et al., 2011; Ribeiro-Silva et al., 2018).

#### Common Hub Genes Between Tissue Pair

**FIGURE 7:**
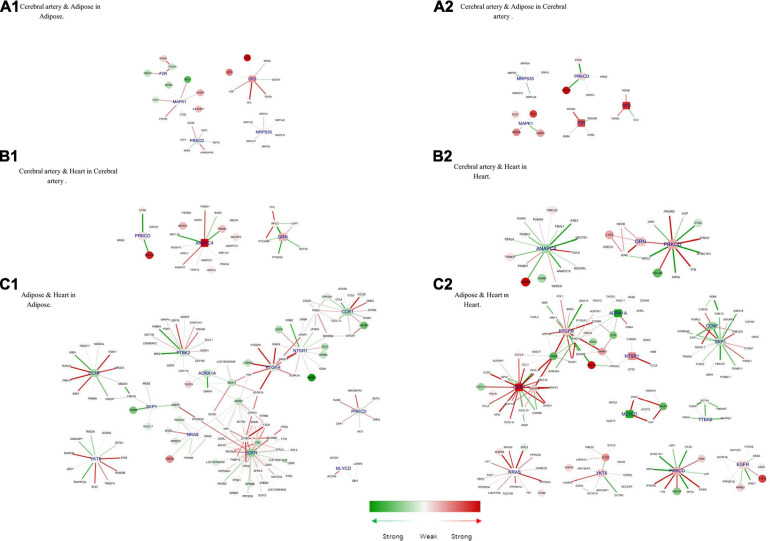
Hub genes of common genes between tissue pair and their corresponding modules. **(A1)** Cerebral artery and adipose in adipose. **(A2)** Cerebral artery and adipose in cerebral artery. **(B1)** Cerebral artery and heart in cerebral artery. **(B2)** Cerebral artery and heart in heart. **(C1)** Adipose and heart in adipose. **(C2)** Adipose and heart in heart. Gene colored in green was differentially expressed between disease and health conditions and was significantly downregulated under disease, while red means upregulated. Edge colored in green means the interaction between its two linked genes was perturbed and was inactivated under disease condition, while red means activated. The width of edges reflect the magnitude of the perturbation. Tissue-specific hub genes were shaped with round rectangle and labeled with blue.

Next, the shared and unique hub genes between the tissue pairs were examined. To achieve this, the common hub genes between tissue pairs were defined as those in *Hub genes of tissue-specific genes*. Based on the common hub genes, their neighbors in the corresponding tissue-specific DN and modules were selected. Thus, we observed the differences and similarities between the same hub genes in different tissues (Figure 7).

Complement factor D (*CFD*), coagulation factor II thrombin receptor (*F2R*), mitogen-activated protein kinase 1 (*MAPK1*), mitochondrial ribosomal protein S35 (*MRPS35*), and *PRKCD* were important in both the adipose tissue and cerebral artery. Consistent with the common biological functions in the heart and adipose tissue, the interaction between *PRKCD* and *F2R* activates the biological process of positive regulation of the MAPK cascade. MAPK1 is closely related to the increased extracellular volume in the cerebral artery and adipose tissue, which is a characteristic in patients with HFpEF (Omori et al., 2020). In the cerebral artery, the interaction between *MAPK1*, *FABP5* (recombinant human fatty acid-binding protein-5), and *FN1* (fibronectin 1) was involved in the biological processes of response to wounding, which may accelerate the process of atherosclerosis (Pintucci et al., 2002). Additionally, *IL6ST* (interleukin 6 signal transducer) uniquely interacted with *MAPK1* in the adipose tissue, which could influence the activation of the JAK-STAT3 pathway and regulate lipid metabolism (Odermatt et al., 2020).

Three common hub genes were identified between the cerebral artery and the heart. The function of these three hub genes is shown in Figure 7A. The hub gene *ANAPC4* (anaphase-promoting complex subunit 4) was significantly upregulated in the artery but downregulated in the heart. Most interactions linked with *ANAPC4* in the heart were inactivated in HFpEF. The expression of *ANAPC4* was closely related to cell cycle in the heart and cerebral artery. The interaction between *ANAPC4* and *FBXL22* (F-box and leucine-rich protein 22) was weak in the heart, which may result in the upregulation of *FBXL22* and impaired contractile function of the heart (Spaich et al., 2012). In the cerebral artery, *ANAPC4* may increase the expression of *PSMB1* (proteasome 20S subunit beta 1), which is closely related to vascular remodeling (Wang et al., 2013). The hub gene *PRKCD* was upregulated in both tissues. Several interactions linked with *PRKCD* were perturbed in the heart of patients with HFpEF but not in the artery. In this study, *PRKCD* was correlated with shared biological processes of the heart and cerebral artery. For example, PRKCD suppresses macro-/microautophagy (Zhang et al., 2017). PRKCD regulates the expression of VEGFR 2 (VEGF receptor 2) and PDGFR-β in arteries and is closely related to collateral vessel formation (Lizotte et al., 2013). However, the interaction between PRKCD and TTR (transthyretin) was enhanced in the heart and may promote the progression of cardiac amyloidosis in patients with HFpEF (Chen et al., 2018). GRN (Granulin) was activated in the cerebral artery and heart, and the interaction between GRN and related genes was enhanced in the heart. The interaction between LYZ2 (Lysozyme C-2) and GRN in the heart is associated with the activation of chronic inflammation (Wang C. et al., 2018). Additionally, GRN may influence the remodeling of the cerebral artery by positively regulating N2 (Niemann–Pick type C2) and inhibiting the activation of the ERK 1/2 MAPK signaling pathway (Csepeggi et al., 2011).

The following 12 hub genes were shared between the adipose tissue and the heart: *ADRA1A* (adrenoceptor alpha 1A), *CCNF* (cyclin F), *CCR1* (C–C motif chemokine receptor 1), *EGFR* (epidermal growth factor receptor), *MLYCD* (malonyl-CoA decarboxylase), *NRAS* (NRAS proto-oncogene, GTPase), *NSTR1* (neurotensin receptor 1), *PRKCD*, *PTGFR* (prostaglandin F receptor), *SKP1* (S-phase kinase-associated protein 1), *TTBK2* (tau tubulin kinase 2), and *YKT6* (*Ykt6 v-Snare* homolog). These hub genes can activate the calcium signaling pathway and promote the dysfunction of the heart and adipose tissue (Huang and Chen, 2008; Li et al., 2008; Ye et al., 2014; Kao et al., 2015; Kuchay et al., 2017; Wang M. et al., 2018; Abe et al., 2020; da Silva Rosa et al., 2020; Esteves et al., 2020; Wu L. et al., 2020). *CCR1*, which was downregulated in the adipose tissue but significantly upregulated in the heart, is directly associated with more than 10 other genes in the two tissues. The major interactions of *CCR1* were significantly activated in the heart but not in the adipose tissue. The interaction between *CCR1* and *MCHR* 1 (melanin-concentrating hormone receptor 1) was upregulated in the adipose tissue, which promoted fat accumulation (Kring et al., 2008). However, the activation of *CCR1* inhibited the expression of *CXCL 11* (C–X–C motif chemokine 11) and consequently alleviated heart inflammation (Ramirez-Carracedo et al., 2020).

#### Modules in the Merged PPI Network

**FIGURE 8:**
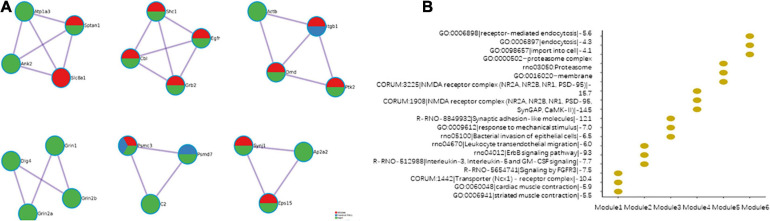
Modules in the merged protein–protein interaction (PPI) network. **(A)** The composition of the modules. **(B)** Function enrichment results of the modules.

The modules effectively indicate the characteristics of the PPI network. The PPI networks of the cerebral artery, heart, and adipose tissue were merged. Six modules were identified in the merged PPI network (Figure 8A). Modules 1, 2, and 6 revealed the interactions between the heart and adipose tissue. Modules 3 and 5 indicate the interactions between the heart, adipose tissue, and cerebral arteries. Module 4 indicated the heart characteristics. The functional enrichment analysis of the six modules revealed the characteristics of HFpEF (Figure 8B). For example, *ITGB1*, *PTK2*, and *ACTB* in module 3 were involved in the biological processes of leukocyte transendothelial migration. This indicated that the migration of proinflammatory factors among these tissues promotes the activation of systemic chronic inflammation.

### Revealing Potential Drug Candidates by Molecular Docking

#### Potential Drug Candidates by Molecular Docking for Housekeeping DEGs and DCGs

**FIGURE 9:**
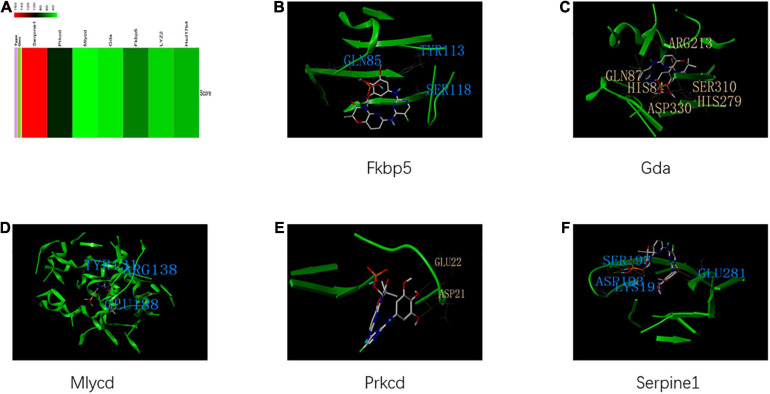
Housekeeping genes and housekeeping interactions. **(A)** The heatmap of score between heart failure with preserved ejection fraction (HFpEF) and genes of housekeeping genes and housekeeping interactions. **(B)** The binding site and interactions between fostamatinib and FKBP *5*. **(C)** The binding site and interactions between fostamatinib and GDA. **(D)** The binding site and interactions between fostamatinib and MLYCD. **(E)** The binding site and interactions between fostamatinib and PRKCD. **(F)** The binding site and interactions between fostamatinib and SERPINE 1.

The identified housekeeping DEGs and DCGs were closely related to the common biological functions of all three tissues. Thus, these genes may be effective therapeutic targets for common biological functions. The query of the CTD database indicated that these genes can be potential therapeutic targets for HFpEF in humans (Figure 9A). Fostamatinib may be the best choice for targeting the housekeeping DEGs and DCGs (Figures 9B–F), as it formed hydrogen bonds with GLN 87, HIS 84, ASP 330, HIS 279, SER 310, and ARG 213 of GDA, which suggested that the binding was strong and that fostamatinib may effectively regulate the activity of GDA. Further studies are needed to elucidate the structures of LYZ2 and HSD17B4. In addition, the docking scores are shown in Supplementary Table 2.

#### Potential Drug Candidates by Molecular Docking for Modules in the Merged PPI Network

The modules of the merged PPI network were the representative biological functions in HFpEF, and the genes of all modules were the drug targets of the representative biological functions. The correlation between the genes of the modules and HFpEF is shown in Supplementary Figure 1A. The structure of some genes has not been elucidated and hence cannot be used to discover potential therapeutic agents through molecular docking. Benzalkonium and capsaicin may be potential drugs for targeting genes in module 1 (Supplementary Figure 1B). The docking scores of ginsenoside Rb1, vandetanib, ergocalciferol, and afatinib were the highest for the genes of module 2 (Supplementary Figure 1C). Dinoprostone and dinoprost tromethamine exhibited strong binding to the gene products of module 3 (Supplementary Figure 1D). Zinc oxide, baclofen, DL-dimyristoylphosphatidylglycerol, and dobutamine can be personalized medicine for targeting the genes of module 4 (Supplementary Figure 1E). Gentamicin and hydroxyethyl starch exhibited good binding to the gene products of module 5 (Supplementary Figure 1F). Adenosine may be an effective drug for targeting the genes in module 6 (Supplementary Figure 1G). Hydrogen bonds were formed between most compounds and genes of modules, which indicated that these compounds may effectively regulate the activity of these genes. In addition, the docking scores are shown in Supplementary Table 2.

#### Potential Drug Candidates by Molecular Docking for the Hub Tissue-Specific Genes

The tissue-specific hub genes were involved in the specific biological functions of tissues. *CPD*, *POLR1C*, *IGFBP7*, *KNG2*, and *CCL27* were closely related to HFpEF in humans (Supplementary Figure 2A). Guanidinoethylmercaptosuccinic acid may effectively target the tissue-specific genes (Supplementary Figures 2B,C), as it formed hydrogen bonds with the gene products. However, the structures of POLR1C, IGFBP7, and KNG2 have not been elucidated, and further studies are needed for the development of personalized medicine for HFpEF. In addition, the docking scores are shown in Supplementary Table 2.

#### Potential Drug Candidates by Molecular Docking for Hub Common Genes

The correlation between the common hub genes and HFpEF is shown in Supplementary Figure 3. Fostamatinib and 13-acetylphorbol exhibited the highest docking scores for the hub genes of adipose tissue and cerebral arteries [Supplementary Figures 3A(b–d)]. Erlotinib, lapatinib, nizatidine, and (2S)-8-[(tert-butoxycarbonyl) amino]-2-(1H-indol-3-yl) octanoic acid are potential drugs for targeting the hub genes of the heart and adipose tissue [Supplementary Figures 3B(b–j)]. Tamoxifen can be a potential drug for targeting the hub genes of the cerebral artery and heart [Supplementary Figures 3C(b–e)]. In addition, the docking scores are shown in Supplementary Table 2.

## Discussion

The progression of HFpEF, a systemic disease, is characterized by the dysfunction of the cerebral artery, adipose tissue, and heart. In this study, the shared and unique pathological features of the cerebral artery, adipose tissue, and heart in HFpEF were investigated at the network, gene, and module levels. Additionally, the correlation between key genes and HFpEF was investigated. Some small-molecule compounds showed beneficial effects on complex diseases (Zhao et al., 2019, 2021; He et al., 2021; Zheng et al., 2020). Furthermore, the Drugbank database and molecular docking were used to identify potential drug targets for HFpEF.

In total, 2,336, 427, and 1,199 DEGs were identified in the heart, cerebral artery, and adipose tissue. The following five housekeeping genes were identified in the three tissues: *MLYCD*, *FKBP5*, *PRKCD*, *SERPINE1*, and *GDA*. Additionally, housekeeping gene interactions, modules in the merged PPI networks, tissue-specific hub genes, and shared hub genes were identified using the multilevel comparative framework. These specific genes indicated the characteristics of HFpEF. Additionally, the results of the CTD database analysis revealed that these genes were closely related to HFpEF in humans, which indicated that these genes are potential therapeutic targets for the development of personalized therapy and precision medicine for HFpEF. A large part of treatment strategies for HFpEF were based on symptoms of HFpEF patients and expert consensus. Although mineralocorticoid antagonist, exercise therapy, and control hypervolemia showed beneficial effects on HFpEF, effective drugs are still lacking. The combination of molecular docking and multilevel comparative frameworks is a potential strategy for the discovery of effective therapy and personalized medicine for HFpEF. Fostamatinib, benzalkonium, capsaicin, baclofen, etc., maybe personalized medicine for HFpEF. Animal experiments will contribute to explore pharmacological functions of these drug on HFpEF, and some prospective clinical trials will help to identify the clinical effects of these drugs.

Functional enrichment analysis of nonspecific genes revealed that the aberrant biological processes in the heart were similar to those in the adipose tissue. Additionally, some biological processes were unique to the cerebral artery. Furthermore, the common biological processes among the three tissues were mainly related to cell cycle and immune response. These findings were consistent with those of previous studies, which demonstrated that cell cycle and immune response are closely related to diastolic dysfunction and impaired cardiac reserves (Roh et al., 2020; Sava et al., 2020). Heart function is related to the flow dynamics of the cerebral artery, which may influence the gene expression in the cerebral artery and increase the incidence of cerebrovascular events (Karahan et al., 2020). Additionally, the cell cycle and immune response are associated with the incidence of cerebrovascular events, which can lead to cardiac dysfunction (Min et al., 2009). Cell cycle and immune response are the main factors contributing to adipose tissue dysfunction, which could increase the level of proinflammatory adipocytokines and consequently contribute to cardiac dysfunction and cerebrovascular events (Packer, 2019). The housekeeping genes and housekeeping gene interactions are involved in these biological functions. This indicated that housekeeping genes may be effective targets for common biological processes. The results of molecular docking revealed that fostamatinib may be a potential drug for these housekeeping genes. Fostamatinib exerts beneficial effects on cell cycle and immune system in other diseases. Further clinical and basic studies are needed to examine the function of adenosine in HFpEF (Gomez-Puerta and Mócsai, 2013). Additionally, six modules were identified in the merged PPI network. The biological functions of these modules can indicate the differential characteristics of HFpEF. The modules were regarded as personalized treatment targets for HFpEF. Molecular docking of these modules will contribute to the discovery of precision medicine and personalized treatment strategies for HFpEF. The DEGs of modules 3 and 5 were identified in all three tissues. The biological processes of modules 3 and 5 were similar to the common biological processes among the three tissues. The binding of dinoprostone and dinoprost tromethamine was strong with the gene products of module 3. However, the effects of these drugs in HFpEF remain unclear. Gentamicin and hydroxyethyl exhibited strong binding with the gene products of module 5. However, further studies are needed to explore the function of these drugs in HFpEF. Modules 1, 2, and 6 comprised DEGs of the heart and adipose tissue. Thus, these modules may be effective targets for blocking the interaction between the heart and the adipose tissue. The major biological functions of module 1 are related to the dysfunction of adipose tissue and impaired heart function (Pachera et al., 2015). Adipose tissue dysfunction also leads to diastolic dysfunction and impaired cardiac reserve. Capsaicin was the most effective drug for targeting module 1. A previous study also indicated that capsaicin could improve muscle function and insulin resistance in an animal model of chronic heart failure (Okajima and Harada, 2008; Wang et al., 2010). However, further studies are needed to examine the beneficial effects of capsaicin on HFpEF. The biological function of module 2 is related to the inflammatory response (Ryzhov et al., 2017; Easter et al., 2020). The systemic proinflammatory state can exacerbate the symptoms of HFpEF (Sani et al., 2018). The docking scores of ginsenoside Rb1, vandetanib, ergocalciferol, and afatinib were high for the gene products of module 2. Ginsenoside Rb1, vandetanib, and ergocalciferol also exerted beneficial effects against inflammatory responses. Further studies are needed to explore the effects of afatinib on the inflammatory response. The genes of module 6 were enriched in steatosis and lipotoxicity (Yazıcı and Sezer, 2017; Mahmod et al., 2018), which are closely related to diastolic dysfunction. Adenosine may be an effective drug for targeting the genes in module 6. Previous studies have suggested that adenosine can improve tissue perfusion and heart function in an animal model of HFpEF (Davila et al., 2019). However, further clinical and experimental studies are needed to identify the function of adenosine. Module 4 comprised only heart-related DEGs. The biological function of module 4 was associated with the immune response. Zinc oxide, baclofen, DL-dimyristoylphosphatidylglycerol, and dobutamine were potential drugs for targeting the genes in module 4. Most of these drugs are reported to exert beneficial effects on the immune response (Zou et al., 2002; Crowley et al., 2015; Liu et al., 2020).

Genes involved in hemostasis, GPCR ligand, cancer-related pathway, chemokine signaling pathway, and membrane trafficking were upregulated in the heart and adipose tissue. Some studies have demonstrated that hemostasis and chemokine signaling pathways play important roles in HFpEF (Glezeva et al., 2015; Pei et al., 2017). The chemokine signaling pathway increases the secretion of inflammatory factors in the adipose tissue, which may exacerbate systemic proinflammatory state and promote the biological process of hemostasis in the heart. The GPCR ligand, cancer-related pathway, and membrane trafficking biological processes can provide novel insights into the role of the heart and adipose tissue in HFpEF. The hub genes of heart and adipose tissue, including *ADRA1A*, *CCNF*, *CCR1*, *EGFR*, *MLYCD*, *NRAS*, *NTSR1*, *PRKCD*, *PTGFR*, *SKP1*, *TTBK2*, and *YKT6*, were also associated with these pathways. The results of molecular docking revealed that erlotinib, lapatinib, nizatidine, and (2S)-8-[(tert-butoxycarbonyl) amino]-2-(1H-indol-3-yl) octanoic acid may be effective drugs for targeting these genes. Most of these drugs are reported to regulate these pathways (Akimoto et al., 2005; Li et al., 2019; Shen et al., 2019). Cell cycle, mitosis, positive regulation of locomotion, adaptive immune system, signaling receptor tyrosine kinases, innate immune system, metabolism of RNA, and chemokine signaling pathways are the shared pathways of the heart and cerebral artery. Previous studies have demonstrated that these pathways are associated with diastolic function and cerebral arterial perfusion. Brain hypoperfusion may induce hypertension, and impaired diastolic function contributes to brain hypoperfusion (Adamski et al., 2018; Walas et al., 2019). The hub genes of the heart and cerebral artery, including *ANAPC4*, *GRN*, and *PRKCD*, play an important role in these pathways. Tamoxifen may be an effective drug for targeting these genes. However, the effects of tamoxifen in HFpEF remain unclear. The common pathways of the adipose tissue and cerebral arteries are associated with cell development, chemotaxis, and apoptosis. A previous study also indicated that fostamatinib regulates cell development, chemotaxis, and apoptosis (Kuiatse et al., 2015). Further studies are needed to examine the role of fostamatinib in HFpEF.

Furthermore, the functional enrichment of all DEGs of the heart, adipose tissue, and cerebral arteries revealed that they are enriched in inflammatory response and cardiac hypertrophy in the heart. These biological processes are important pathophysiological mechanisms in HFpEF (Wu et al., 2001; Xuan et al., 2020). The enrichment analysis of tissue-specific genes also highlighted that immune response was significantly enriched in heart tissues, which is closely related to the inflammatory response and cardiac hypertrophy (Adzika et al., 2019). Some studies have also demonstrated that the heart-specific hub genes, including *IGFBP7*, *CCL27*, and *KNG2*, are involved in the immune response. Guanidinoethylmercaptosuccinic acid was an effective drug for targeting these genes. A previous study also indicated that guanidinoethylmercaptosuccinic acid could regulate the immune response *in vitro* (Lynch et al., 1988). Further studies are needed to examine if guanidinoethylmercaptosuccinic acid can exert similar beneficial effects in HFpEF. These results indicated that the response to peptides and regulation of cell adhesion are specific biological processes in the adipose tissue. Previous studies have demonstrated that the response to peptides is related to lipid metabolism and influences the regulation of cell adhesion, which is associated with the prognosis of HFpEF. The function enrichment analysis of tissue-specific genes indicated that lipid metabolism is enriched in the adipose tissue. The effect of CPD and guanidinoethylmercaptosuccinic acid on lipid metabolism is not clear. Gene expression (transcription) was enriched in the cerebral arteries. Furthermore, the functional enrichment analysis of tissue-specific genes revealed similar biological processes. *POLR1C* also exhibited related biological functions in a previous study (Gauquelin et al., 2019). The structure of POLR1C requires further investigation.

## Conclusion

The multitissue and multilevel comparative analyses provided novel insights into the pathogenesis of HFpEF. *MLYCD*, *FKBP5*, *PRKCD*, *SERPINE1*, and *GDA* are housekeeping genes in the adipose tissue, heart, and cerebral arteries. The aberrant biological processes in the heart were similar to those in the adipose tissue. Cell cycle and immune system were common biological processes of the adipose tissue, heart, and cerebral arteries. Hemostasis, GPCR ligand, cancer-related pathway, chemokine signaling pathway, and membrane trafficking were significantly enriched in the heart and adipose tissue. Cell cycle, mitosis, positive regulation of locomotion, adaptive immune system, signaling receptor tyrosine kinases, innate immune system, metabolism of RNA, and chemokine signaling pathway were the common pathways of the heart and cerebral artery. The common pathways of the adipose tissue and cerebral artery were associated with cell development, chemotaxis, and apoptosis. Inflammatory response, cardiac hypertrophy, and immune response were enriched in the heart. Lipid metabolism is a specific biological function of the adipose tissue. Furthermore, gene expression (transcription) was enriched in the cerebral artery. Potential therapeutic agents for HFpEF were investigated using molecular docking.

## Data Availability Statement

All data used or analyzed in this study are available upon request by contacting the corresponding authors.

## Ethics Statement

The animal study was reviewed and approved by Animal Ethics Committee of Shandong University of Traditional Chinese Medicine (Ethics No. SDUTCM2018071501).

## Author Contributions

SS and XL designed the study. SS performed the computation. GZ, SS, and QY analyzed the results and wrote the manuscript. YW revised the manuscript. All authors read and approved the submitted version of the manuscript.

## Conflict of Interest

The authors declare that the research was conducted in the absence of any commercial or financial relationships that could be construed as a potential conflict of interest.

## Footnotes

1 http://www.ncbi.nlm.nih.gov/geo/

2 http://ctdbase.org/

3 https://string-db.org/

4 https://www.drugbank.ca/

5 http://www.rcsb.org/
