# Supplementary figures and images for: Multiple-Tissue and Multilevel Analysis on Differentially Expressed Genes and Differentially Correlated Gene Pairs for HFpEF
Source: Front Genet. 2021 Jul 8;12:668702. doi: 10.3389/fgene.2021.668702 (PMC8296822; doi:10.3389/fgene.2021.668702)

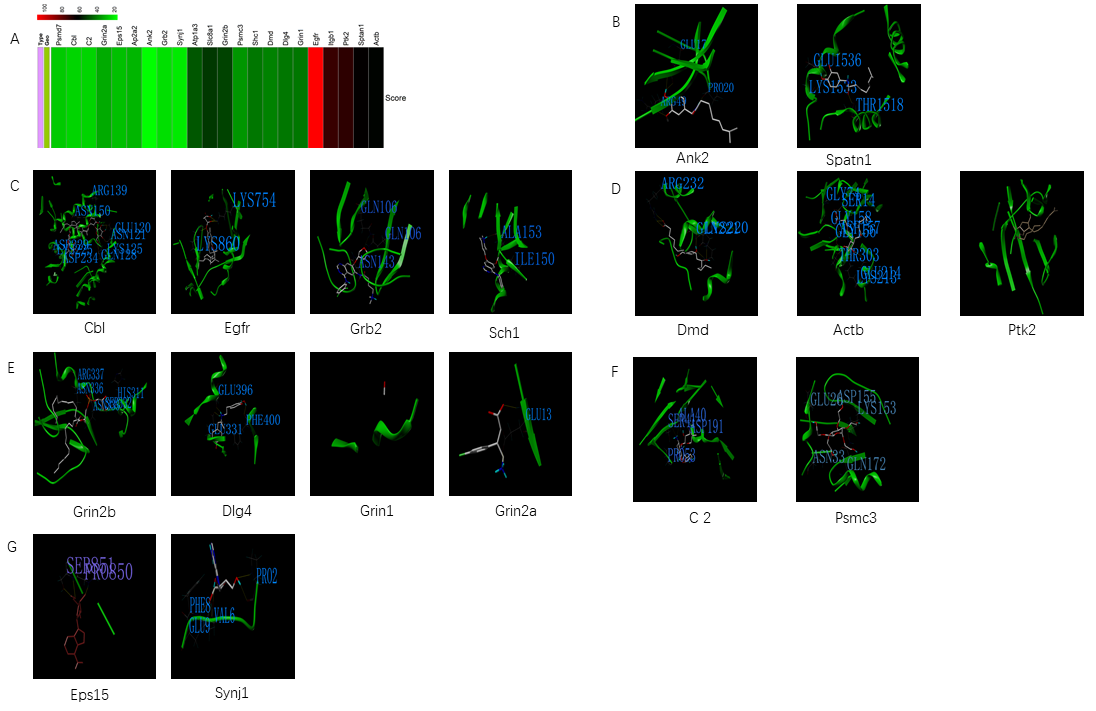

Supplement: Supplementary file 1 [file Image_1.TIF]

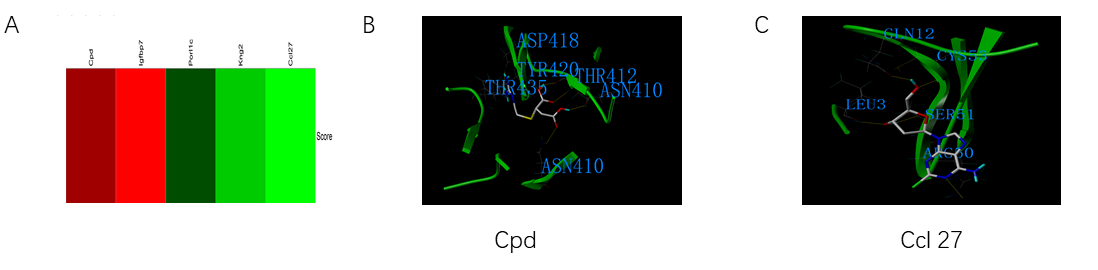

Supplement: Supplementary file 2 [file Image_2.TIF]

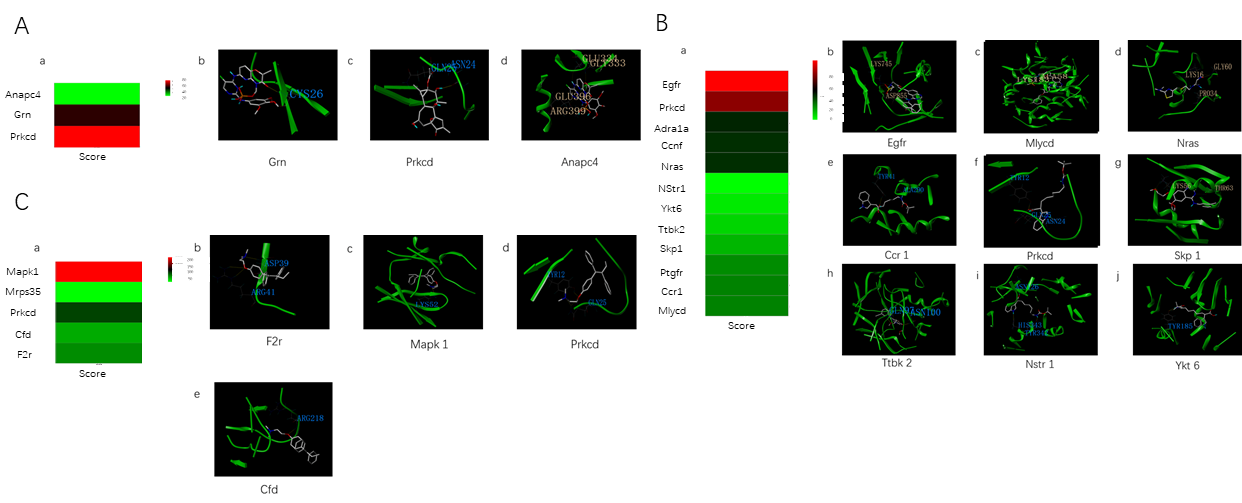

Supplement: Supplementary file 3 [file Image_3.TIF]
