# Supplementary material for: Multiple-Tissue and Multilevel Analysis on Differentially Expressed Genes and Differentially Correlated Gene Pairs for HFpEF
Source: Front Genet. 2021 Jul 8;12:668702. doi: 10.3389/fgene.2021.668702 (PMC8296822; doi:10.3389/fgene.2021.668702)
Supplement: Supplementary file 4 [file Table_1.DOCX]

Supplementary Table 1 Characteristics of three tissue-specific differential networks

| Characteristics  Tissues | Clustering coefficient | Number of nodes | Network density | Network centralization | Network heterogeneity | Characteristic path length | Avg. Number of neighbors |
| --- | --- | --- | --- | --- | --- | --- | --- |
| Cerebral arteries | 0.051 | 360 | 0.006 | 0.092 | 1.667 | 6.569 | 2.267 |
| Heart | 0.105 | 1477 | 0.002 | 0.037 | 1.443 | 8.092 | 2.978 |
| Adipose tissue | 0.077 | 995 | 0.003 | 0.042 | 1.543 | 7.831 | 2.718 |
