# Supplementary material for: Multiple-Tissue and Multilevel Analysis on Differentially Expressed Genes and Differentially Correlated Gene Pairs for HFpEF
Source: Front Genet. 2021 Jul 8;12:668702. doi: 10.3389/fgene.2021.668702 (PMC8296822; doi:10.3389/fgene.2021.668702)
Supplement: Supplementary file 5 [file Table_2.DOCX]

Supplementary Table 2 Docking scores

| Protein | Molecule | Score |
| --- | --- | --- |
| Mlycd | Fostamatinib | 5.38 |
| Fkbp 5 | Fostamatinib | 5.72 |
| Serpine 1 | Fostamatinib | 2.36 |
| Gda | Fostamatinib | 7.44 |
| Prkcd | Fostamatinib | 1.71 |
| Sptan 1 | Benzalkonium | 6.21 |
| Ank2 | Capsaicin | 4.2718 |
| Cbl | Ginsenoside Rb1 | 10.9753 |
| Shc1 | Vandetanib | 6.2097 |
| Egfr | ergocalciferol | 7.4191 |
| Grb2 | Afatinib | 8.157 |
| Dmd | Dinoprostone | 7.0263 |
| Ptk2 | Dinoprostone | 9.4714 |
| Actb | Dinoprost tromethamine | 11.5347 |
| Grin2b | DL-dimyristoylphosphatidylglycerol | 10.7345 |
| dlg4 | Dobutamine | 8.0563 |
| Grin2a | Baclofen | 3.6755 |
| Grin1 | Zinc oxide | 3.2646 |
| Psmc3 | Gentamicin | 7.1997 |
| C2 | Hydroxyethyl Starch | 5.3894 |
| Synj1 | Adenosine | 4.1852 |
| Eps1 | Adenosine | 1.4688 |
| Cpd | Guanidinoethylmercaptosuccinic acid | 5.54 |
| Ccl27 | Guanidinoethylmercaptosuccinic acid | 3.25 |
| Prkcd | 13-Acetylphorbol | 2.01 |
| Grn | Fostamatinib | 2.09 |
| Anapc4 | Fostamatinib | 5.34 |
| Prkcd | (2S)-8-[(tert-butoxycarbonyl) amino]-2-(1H-indol-3-yl) octanoic acid | 4.23 |
| Ccr2 | (2S)-8-[(tert-butoxycarbonyl) amino]-2-(1H-indol-3-yl) octanoic acid | 9.16 |
| Mlycd | Lapatinib | 8.93 |
| Nras | Nizatidine | 7.98 |
| Ntsr1 | (2S)-8-[(tert-butoxycarbonyl) amino]-2-(1H-indol-3-yl) octanoic acid | 8.75 |
| Egfr | Lapatinib | 7.87 |
| Skp1 | Erlotinib | 7.19 |
| Ttbk2 | Erlotinib | 8.08 |
| Ykt6 | (2S)-8-[(tert-butoxycarbonyl) amino]-2-(1H-indol-3-yl) octanoic acid | 6.39 |
| Cfd | Tamoxifen | 4.8 |
| F2r | Tamoxifen | 3.32 |
| Mapk1 | Tamoxifen | 5.64 |
| Prkcd | Tamoxifen | 4.43 |
